# Rupture of urinary bladder diverticulum

**DOI:** 10.11604/pamj.2021.39.171.29742

**Published:** 2021-07-06

**Authors:** Danilo Coco, Silvana Leanza

**Affiliations:** 1 Department of General Surgery, Ospedali Riuniti Marche Nord, Pesaro, Italy,; 2 Department of General Surgery, Carlo Urbani Hospital, Jesi, Ancona, Italy

**Keywords:** Urinary bladder diverticulum, patient, male

## Image in medicine

**Figure 1:**
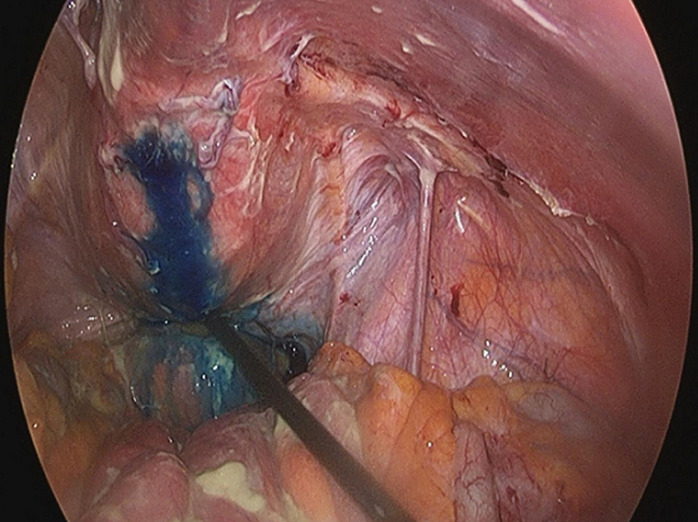
methylene blue to the area through the bladder catheter

Patient was a 61-year-old Caucasian male. He was admitted to the hospital with non-specific symptoms, including fever, vomiting, general weakness, painful abdominal distension, constipation, decreased urine output and voiding complaints. Medical history included arterial hypertension and depressive disorders which had caused the patient to neglect periodic urology check-ups. He reported a recent hospitalization with uroperitoneum which was treated conservatively with a bladder catheter. His temperature was 38°C and a blood pressure calculation of 90/62 mmHg, heart rate of 120 regular beats/min, and respiratory rate of 30 breaths/min were recorded. Laboratory testing revealed a hemoglobin count of 10.8 g/dL, serum creatinine of 2.7 mg/dL, white blood cells (WBC) to 19,300/μL and a C-reactive protein (CRP) level of 300.2 mg/dL. Physical examination revealed diffuse pain and tenderness associated with rebound pain, particularly in the hypogastric area. Computerized tomography (CT) scan confirmed the presence of a small amount of free fluid in the abdominal cavity, causing excessive fluid accumulation in a small and not empty bladder, a small amount of free air in the abdomen. Patient was transferred to the operating theater. We introduced methylene blue to the area through the bladder catheter. This method demonstrated a large spread of methylene blue in the peritoneal cavity (Figure 1). An intraperitoneal rupture of the large bladder diverticulum was discovered, located on the right superolateral wall of the bladder. We performed peritoneal lavage and bladder raffia with interrupted 2/0 Vicryl sutures by laparoscopy, leaving drains in place. The patient was discharged from the hospital on the 6^th^ postoperative day.

